# Richmond Academy of Medicine and Science

**Published:** 1898-08

**Authors:** 


### Richmond Academy of Medicine and Science.

MEETING HELD JULY 12, 1898.

Dr. M. D. Hoge, Jr., President, in the chair. Dr. Mark W. Peyser, secretary and reporter.

Dr. George Ben Johnston reported some abdominal cases.

Dr. Jacob Michaux exhibited casts of the palms of the hands and soles of the feet of a young man, aged nineteen years. The patient had a fever, 102°, the nature of which was indefinite. The patient was spare built. There was no eruption nor tongue symptoms. He was not seen until three days after the inception of the fever. A dose of three grains of quinine was given and three hours after there was almost a convulsion, though consciousness was retained. In four or five hours a rash appeared. Dr. Michaux said he would have been uncertain as to the influence of the quinine and its dose producing the exfoliation were it not for the information derived from the mother, an intelligent woman, that it had occurred before, but she had neglected to mention it. He had heard of but one other case. The casts in his came off in a week, and the whole epidermis of the body was shed in particles. This explanation of the phenomenon was idiosyncrasy, and a rather marked case was that of Dr. Bolton, who, whenever he uncorked a bottle of morphine, although holding it out at arm's length, would have an eruption to appear all over the body. The first time the effect was produced was when weighing out a half-grain, a sunburnt appearance was noticed around the eyes. The cause was not suspected for some time.

Dr. J. N. Upshur said the history of Dr. Michaux's case was more like scarlet fever than anything else. He did not think the mother's information amounted to anything, for that kind derived from relatives of patients was unreliable. There was nothing in the physiological action of quinine to explain the condition. The period of incubation was that of scarlet fever, and the explanation thus was more natural than by quinine.

Dr. Michaux asked leave to state that he had again given a like dose of quinine with a like result. The information obtained from the mother was given her by an intelligent physician who had attended the patient in time past.

Dr. Jonston said he believed Dr. Michaux's explanation of the case, the correct one, viz., idiosyncrasy. He had never seen such a profound effect from quinine, but had seen a severe dermatitis. The following confirmed his belief. In the case of an old lady, a five-grain dose of iodide of potassium produced alarming symptoms. Two and a-half grains produced the same, and likewise did continued reductions, even until one-tenth of a grain was reached, when there were the same symptoms, with same degree of violence. Dr. J. B. McCaw will faint when he smells camphor. Dr. Bryant brought a case to him for operation, and he was about to pack with iodoform gauze, when the doctor asked for time to leave the room, saying if he remained until the container was opened he would have nettlerash before he could reach the bottom of the stairs. Dr. Morris, of this city, cannot pass within seven feet of growing poison-oak without having its characteristic effect. All these being so, why could not quinine produce the effect shown by Dr. Michaux? He was prepared to believe it true.

Dr. W. S. Beazley exhibited three teeth extracted from the mouth of an infant, the first when it was thirteen days old, the second on the fifteenth day, and the third on its nineteenth day. He saw the infant on the third day after birth, and found the left cheek and eye and the nose inflamed, the last two also discharging. In examining the jaw later he saw an opening in the gum from which pus was exuding, and also a loose tooth, which he pulled, two days later a molar was seen, which was pulled, and again in four days a second molar, which met the same fate. All the teeth came from the left upper jaw. He was told there was no evidence of teeth at birth.

